# A novel function of short cationic peptide FP-CATH9 without antimicrobial activity reverses resistance to minocycline in common multidrug-resistant gram-negative bacteria

**DOI:** 10.1128/spectrum.02908-24

**Published:** 2025-02-25

**Authors:** Yingqi Tang, Jiye Liu, Jiani Yan, Zhixiong Xie, Lipeng Zhong

**Affiliations:** 1 Center for Molecular Diagnosis and Precision Medicine, The First Affiliated Hospital, Jiangxi Medical College, Nanchang University, Nanchang, China; 2 School of Economics and Management, Nanchang University 47861 , Nanchang, Jiangxi, China; 3 Jiangxi Provincial Center for Advanced Diagnostic Technology and Precision Medicine, The First Affiliated Hospital, Jiangxi Medical College, Nanchang University, Nanchang, China; 4 Hubei Key Laboratory of Cell Homeostasis, College of Life Sciences, Wuhan University, Wuhan, China; 5 School of Public Health, Nanchang University 47861 , Nanchang, Jiangxi, China; Seton Hall University, South Orange, New Jersey, USA

**Keywords:** short cationic peptide, minocycline, gram-negative drug-resistant bacteria

## Abstract

The increase in bacterial resistance to minocycline and other tetracyclines poses a serious threat to global public health. Because the development of new antibiotics has proven problematic, antibiotic sensitization therapy is now an effective coping strategy. While antimicrobial peptides generally exhibit broad-spectrum antibacterial activity and good biocompatibility, naturally truncated portions of antimicrobial peptides (such as snake cathelicidin) often do not exhibit antimicrobial activity, and their function remains unknown. FP-CATH9 is a short cationic peptide derived from FP-CATH (snake cathelicidin antimicrobial peptide) with an amphiphilic α-helical structure and no discernible antibacterial activity. However, FP-CATH9 was previously found to significantly enhance the activity of minocycline against gram-negative bacteria. In the present paper, clinically relevant minocycline-resistant gram-negative bacteria (*Escherichia coli*, *Klebsiella pneumoniae*, *Acinetobacter baumannii*, and *Pseudomonas aeruginosa*) were used as test bacteria for antibiotic sensitization screening. The sensitization activity of FP-CATH9 was found to be dose dependent in a double-dilution assay. The synergistic activity of FP-CATH9 on minocycline was subsequently determined using the checkerboard method. An ethidium bromide efflux test revealed that FP-CATH9 caused an accumulation of minocycline in bacteria. Additionally, FP-CATH9 exhibited low hemolytic activity on red blood cells and low cytotoxicity on Raw264.7 cells. In an *in vivo* model of bacterial infection, FP-CATH9 combined with minocycline exhibited an 80% protective effect on *Galleria mellonella* larvae infected with multidrug-resistant *K. pneumoniae*. In summary, FP-CATH9 is a new antibiotic adjuvant that reverses the resistance of gram-negative bacteria to minocycline by increasing intracellular accumulation of minocycline. This finding has broad application potential.

**IMPORTANCE:**

The existence of the efflux pump system enables bacteria to expel antibiotics, reduce the concentration of antibiotics in cells, and make antibiotics unable to effectively inhibit or kill bacteria, which is one of the main mechanisms of bacterial resistance to antibiotics. However, some efflux pumps are substrate specific, while others are with a wide range of substrates. In this study, FP-CATH9 as a new antibiotic adjuvant can specifically reverse the resistance of gram-negative bacteria to minocycline by increasing the intracellular accumulation of minocycline in bacteria and provides a new way to solve the increasing problem of bacterial drug resistance.

## INTRODUCTION

While established antibiotics have previously achieved remarkable results in the treatment of bacterial infections, microbial resistance to these antibiotics has become increasingly strong in recent years (1, 2). Highlighting the severity of the issue, the World Health Organization has listed several multidrug-resistant gram-negative bacterial strains, specifically the carbapenem-resistant strains, as key priority pathogens, because these strains are resistant to last-line antibiotics and may spread drug resistance to other bacteria. The problem of microbial resistance is already a serious threat to human health, and the number of people who die each year from infections is only expected to increase (3, 4). Currently, around 700,000 people die each year from drug-resistant diseases, but this number could rise to 10 million a year by 2050, which is more than the worldwide incidence of cancer deaths each year (5).

Despite this pressing need for new antimicrobial agents, the development of new antibiotics has proven especially difficult, and no original antibiotic has reached the market in nearly 30 years. The antibiotic development pipeline typically includes compound screening, clinical trials, regulatory approval, and numerous other costs, requiring a great deal of investment. In general, pharmaceutical companies are more interested in investing in high-yield drugs, and antibiotic development is considered unattractive, especially because market saturation prospects and margins are considered poor. Moreover, the frequency of repeat detection of antibiotics of known structures is continually increasing, and new antibiotic classes are becoming more and more difficult to find. While new antibiotic development is sluggish, antibiotic sensitizers have shown themselves to be promising antibacterial adjuvants, even though they exhibit poor or no antibacterial effect when used alone. Trimethoprim is a prime example, and when used in combination with antibiotics, it has a clear synergistic effect, increasing the effects of co-administered antibiotics several times to several hundreds of times.

Tetracycline class antibiotics play an antibacterial role by inhibiting bacterial protein synthesis, and they include chlortetracycline, tetracycline, and minocycline (6). Of these, minocycline exhibits several advantages, including strong tissue permeability, high bioavailability, long half-life, metabolites that exhibit antibacterial activity, and good tolerance in patients with renal insufficiency (7). However, *P. aeruginosa* is naturally resistant to tetracyclines, and minocycline resistance rates in *K. pneumoniae* and *A. baumannii* increase each year (8). Hence, tetracycline resistance limits the clinical use of this class of antibiotics.

Antimicrobial peptides (AMPs) are small polypeptide molecules that can kill bacteria, fungi, viruses, and other pathogenic microorganisms (9). As a general rule, natural antimicrobial peptides have a low molecular weight, a simple structure, strong bactericidal activity, are non-toxic to mammalian cells and tissues (without residual problems), and have good biocompatibility (10, 11). While naturally processed fragments of these antimicrobial peptides usually exhibit no antimicrobial activity, the precise function of these truncated peptides has not been fully clarified (12). An interesting antimicrobial peptide that exhibits broad-spectrum antibacterial activity is FP-CATH, a novel antimicrobial peptide found in traditional Chinese medicine obtained from *Deinagkistrodon acutus* (13). Interestingly, the FP-CATH9 peptide derived from FP-CATH was found to exert a significant sensitizing effect on minocycline antibiotic during sensitization screening experiments. Because FP-CATH9 does not exhibit antibacterial activity itself, we initially considered the possibility that FP-CATH9 may trigger the production of minocycline. We also investigated the ubiquity of FP-CATH9.

In the present study, clinical minocycline-resistant gram-negative bacteria were isolated and used to evaluate the sensitizing and synergistic effects of FP-CATH9 on minocycline. Additionally, the toxicity and side effects of FP-CATH9 and its combination with minocycline were evaluated in cell biology experiments. Subsequently, the protective effects of FP-CATH9, combined with minocycline, were investigated using the *Galleria mellonella* infection model. By investigating bacterial membrane integrity and minocycline accumulation, the mechanism of FP-CATH9 sensitization of minocycline was also elucidated. In summary, we have investigated a potential combination therapy for infections caused by multidrug-resistant minocycline-resistant gram-negative bacteria, laying a theoretical foundation for peptide-sensitized antibiotic therapy.

## RESULTS

### Analysis of secondary structure of FP-CATH9 using circular dichroism

**Fig 1:**
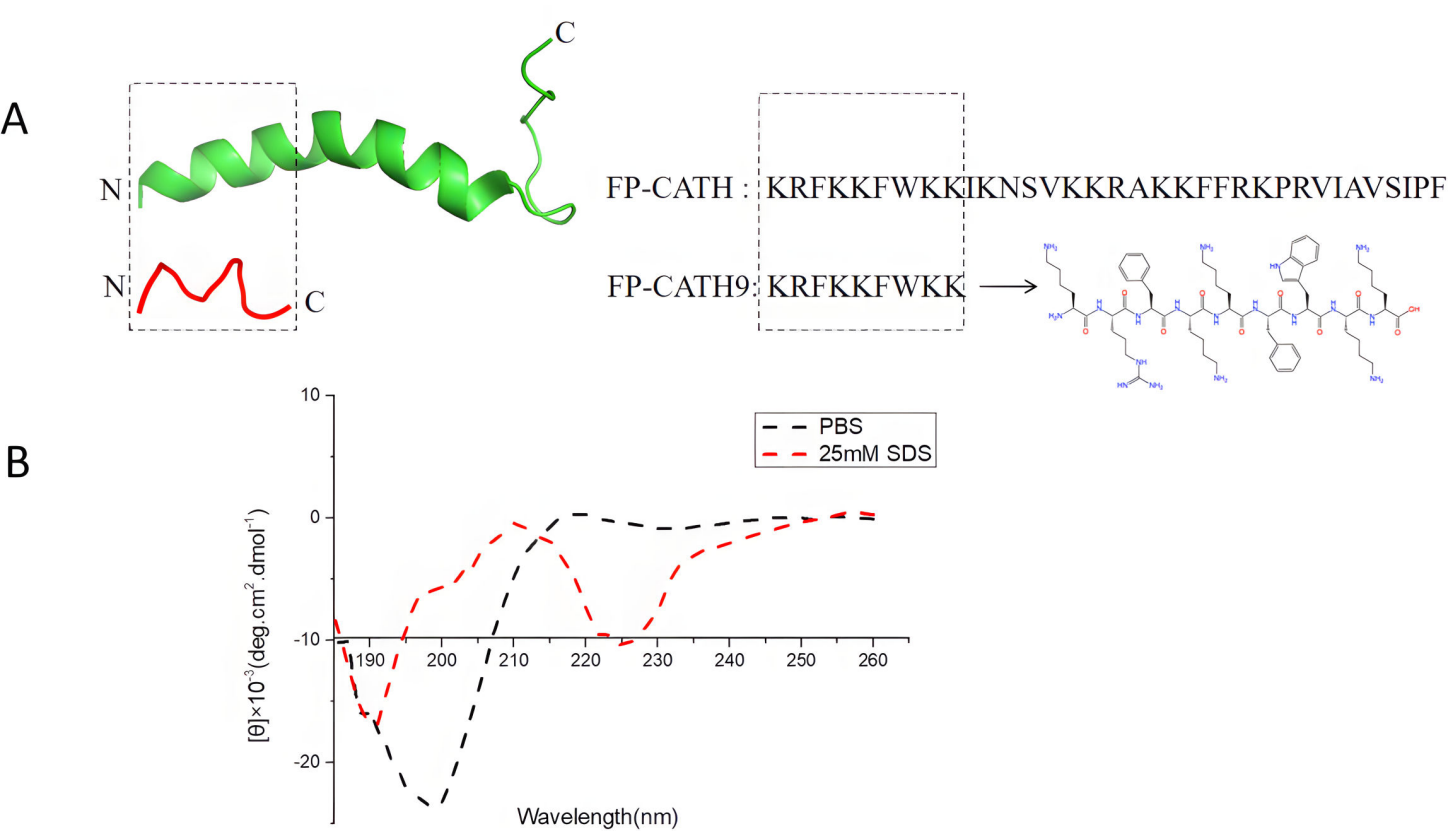
Amino acid sequence and secondary structure of FP-CATH9. (**A**) Amino acid sequence of FP-CATH9 and FP-CATH, (**B**) Circular dichroism diagram of FP-CATH9. The black line represents the membrane simulation condition, and the red line represents the aqueous-phase condition.

FP-CATH9 is derived from the N-terminal nine amino acids of FP-CATH (Fig. 1A). In PBS buffer, FP-CATH9 (^1^KRFKKFWKK^9^, Mw = 1,295.62, pI = 11.84) at a concentration of 10 µM exhibited a random coil structure. Conversely, FP-CATH9 exhibited an atypical helix structure under simulated membrane conditions (using 25 mM SDS) (Fig. 1B).

### Evaluation of antimicrobial activity of FP-CATH9

The antimicrobial activity of FP-CATH9 was determined through broth microdilution and plate assays in an antibiotic disk diffusion test. FP-CATH9 demonstrated no antibacterial activity against the bacteria tested when administered alone (Table 1).

**TABLE 1:** Antimicrobial activity of FP-CATH9^*a*^

Strain	FP-CATH9 MIC (μg/mL)	Inhibition zone (mm)
*E. coli* ATCC BW25113	>512	6
*P. aeruginosa* ATCC 27853	>512	6
*K. pneumoniae* ATCC 700603	>512	6
*A. baumannii* ATCC 19606	>512	6
*S. aureus* ATCC 29213	>512	6
Clinical *P. aeruginosa* 1 (minocycline resistance)	>512	6
Clinical *P. aeruginosa* 2 (minocycline resistance)	>512	6
Clinical *P. aeruginosa* 3 (minocycline resistance)	>512	6
Clinical *K. pneumoniae* 1 (minocycline sensitive)	>512	6
Clinical *K. pneumoniae* 2 (minocycline mediated)	>512	6
Clinical *K. pneumoniae* 3 (minocycline resistance)	>512	6
Clinical *A. baumannii* 1 (minocycline sensitive)	>512	6
Clinical *A. baumannii* 2 (minocycline mediated)	>512	6
Clinical *A. baumannii* 3 (minocycline resistance)	>512	6

^*a*^ The experiment was repeated three times.

### Screening of FP-CATH9 for antimicrobial sensitization of antibiotics

Using the drug-resistant *K. pneumoniae* strain 3 for testing purposes, FP-CATH9 was found to demonstrate sensitizing activity only upon co-administration with minocycline and tigecycline (and not upon co-administration with the other antibiotics listed in Table 2). FP-CATH9 (20 µg peptide in 10 µL) sensitization increased the inhibitory zone of minocycline from 6 to 15 mm, and it increased the inhibitory zone of tigecycline from 16 to 18 mm. The results are summarized in Table 2.

**TABLE 2:** Sensitizing effect of FP-CATH9 on *K. pneumoniae* strain 3^*a*^*^,^*^*b*^

Antibiotic	Inhibition zone (mm) Normal saline control	Inhibition zone (mm) Contains FP-CATH9
IPM	9.06 ± 0.04	9.03 ± 0.05
MEM	8.16 ± 0.12	8.1 ± 0.08
CIP	6	6
SXT	10.23 ± 0.17	10.13 ± 0.12
MH	6.1 ± 0.08	**15.13 ± 0.12**
TGC	16.1 ± 0.08	18.1 ± 0.08
AK	6	6
CRO	6	6
CAZ	6	6
GN	6	6
AMP	6	6
TZP	6	6

^*a*^ AK, amikacin; AMP, ampicillin; CAZ, ceftazidime; CIP, ciprofloxacin; CRO, ceftriaxone; GN, gentamicin; IPM, imipenem; MEM, meropenem; MH, minocycline; SXT, sulfamethoxazole; TGC; tigecycline; TZP, piperacillin-tazobactam.

^*b*^ The experiment was repeated three times.

### FP-CATH9 antibacterial and sensitizing activity to minocycline

**Fig 2:**
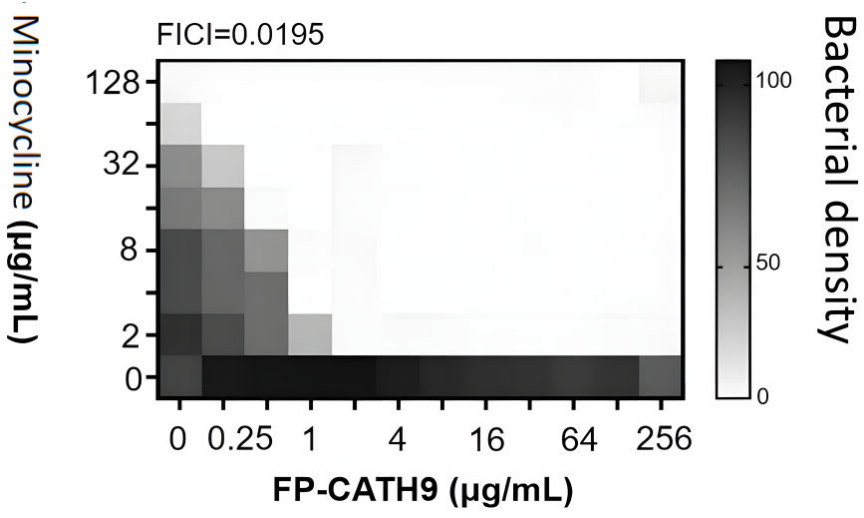
Checkerboard assay to detect the synergistic effect of FP-CATH9 and minocycline. Multidrug-resistant *K. pneumoniae* as a test organism. The experiment was repeated three times, and the results were presented only once.

Against multidrug-resistant *K. pneumoniae*, the MIC of minocycline alone was 128 µg/mL. In comparison, the MIC of minocycline combined with FP-CATH9 was 2 µg/mL, indicating that FP-CATH9 facilitated a 64-fold reduction in the MIC. Additionally, the combined drug susceptibility of minocycline and FP-CATH9 was tested using the checkerboard method. Here, the fractional inhibition concentration index (FICI) index was used to assess whether there was a synergistic effect when these antibacterial drugs were used in combination; a FICI value of ≤0.5 indicates that the antimicrobial combination is synergistic. As shown in Fig. 2, a FICI index of 0.0195 was obtained for FP-CATH9 and minocycline, indicating a strong synergy when used in combination.

### Evaluation of FP-CATH9 sensitizing activity with minocycline using the antibiotic disk diffusion test

**Fig 3:**
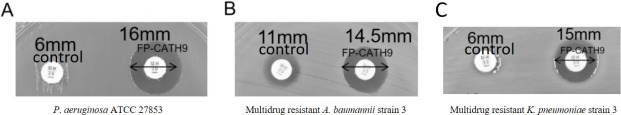
Determination of the synergistic effect of FP-CATH9 and minocycline by plate assay. (**A**) *P. aeruginosa* ATCC 27853, (**B**) multidrug-resistant *A. baumannii* strain 3, and (**C**) multidrug-resistant *K. pneumoniae* strain 3. Control: contains 10 µL normal saline + MH, FP-CATH9: peptide dissolution by 10 µL normal saline (peptide contained 20 µg) + MH.

Against *P. aeruginosa* ATCC 27853, FP-CATH9 exhibited extremely potent sensitizing activity when co-administered with minocycline (Fig. 3A). FP-CATH9 also exhibited extremely strong sensitizing activity against the clinically relevant *A. baumannii* strain 3 (also minocycline resistant) when co-administered with minocycline (Fig. 3B). Together, these results provide firm evidence that FP-CATH9 exhibits sensitizing activity when used in combination with minocycline.

### Determination of bactericidal rate of FP-CATH9 in combination with minocycline

**Fig 4:**
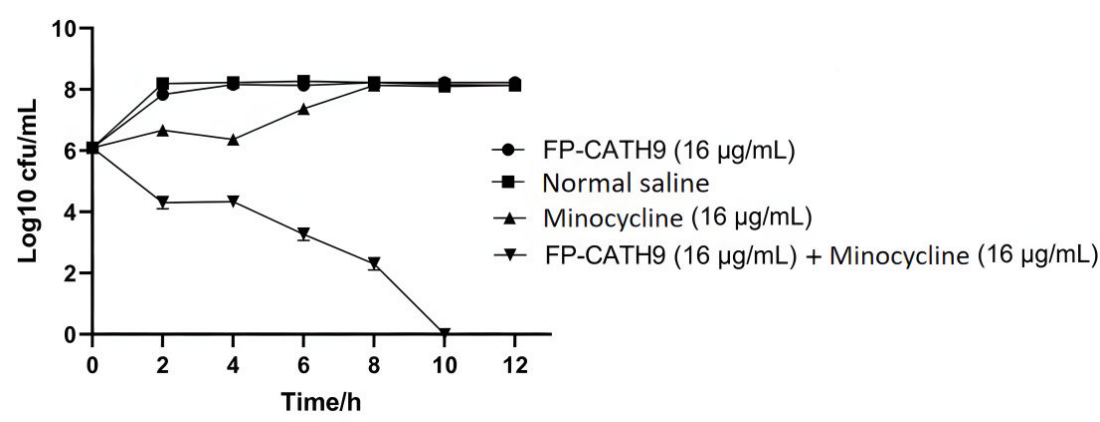
Bacteria time-killing curve with FP-CATH9 and minocycline. Multidrug-resistant *K. pneumoniae* strain 3 as a test organism.

After 4 h of treatment with minocycline only (16 µg/mL), drug-resistant bacterial cultures were again observed to be growing rapidly. After 10 h of treatment with either FP-CATH9 (16 µg/mL) only or minocycline (16 µg/mL) only, growth rates were restored to those of the control saline group, indicating that these treatments had no inhibitory effect. However, co-administration of FP-CATH9 (16 µg/mL) and minocycline (16 µg/mL) completely abolished bacterial growth at 10 h. These results are shown in Fig. 4.

### Determination of hemolytic activity and cytotoxicity of FP-CATH9

**Fig 5:**
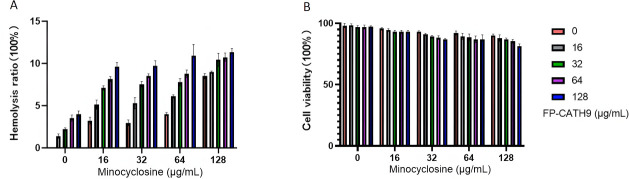
(**A**) The hemolytic activity of different concentrations of FP-CATH9 and minocycline combinations (128–0 µg/mL) on bovine erythrocytes was determined. (**B**) CCK8 assay was used to detect the cytotoxicity of different concentrations of FP-CATH9 and minocycline combinations (128–0 µg/mL) on Raw264.7 cells.

FP-CATH9 exhibited low hemolysis activity against red blood cells (3.97% ± 0.42% at a concentration of 128 µg/mL) when applied in isolation, increasing to 11.33% ± 0.45% when applied in combination with minocycline. As shown in Fig. 5A, these concentrations are far above their effective bactericidal concentration. Additionally, FP-CATH9 exhibited low cytotoxicity against Raw264.7 cells (97.27% ± 0.67% at a concentration of 128 µg/mL), decreasing to 81.43% ± 0.69% when applied in combination with minocycline (Fig. 5B). Together, these results provide evidence that FP-CATH9 exhibits good biocompatibility.

### Serum and thermal stability of FP-CATH9

**Fig 6:**
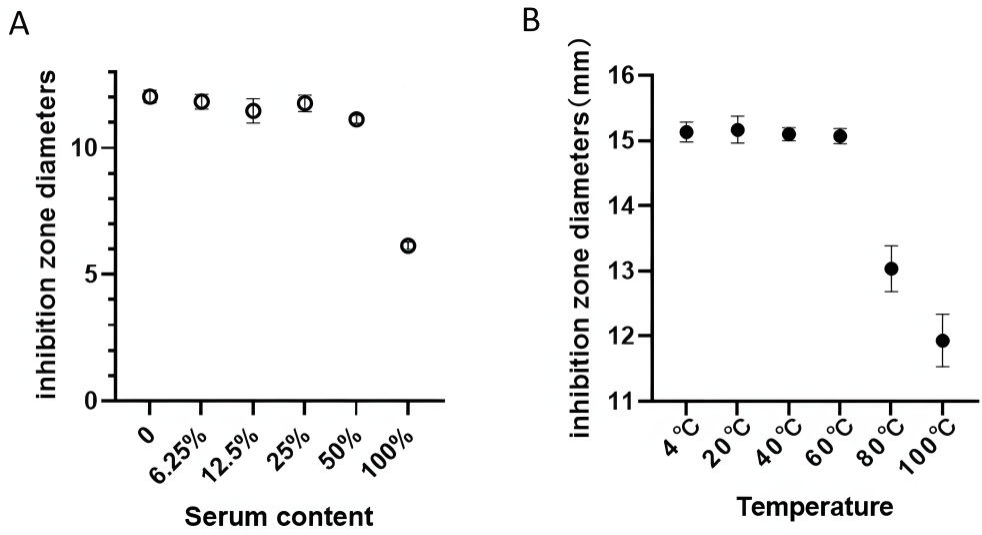
(**A**) Effects of different fetal bovine serum on the sensitization of FP-CATH9 to minocycline. A total volume of 10 µL was added, and a final content of FP-CATH9 of 10 µg was maintained. (**B**) Effects of different temperature treatments on the sensitization of FP-CATH9 to minocycline. A total volume of 10 µL was added, and a final content of FP-CATH9 of 20 µg was maintained. Multidrug-resistant *K. pneumoniae* strain 3 was used as a test organism.

The effects of fetal bovine serum (FBS) and thermal stability on FP-CATH9 were subsequently determined. The influence of FBS on the sensitizing activity of FP-CATH9 was determined using the plate method by comparing changes in the sizes of the inhibition zones between the FBS group, the saline group, and the FBS dilution group (Fig. 6A). While the enhancement effect was slightly reduced when the ratio of FP-CATH9 (C:C) was 1:1, the inhibition zone was still markedly increased (11 mm vs 6 mm for the blank group). The thermal tolerance of FP-CATH9 was determined by comparing changes in the sizes of the inhibition zones after different temperature treatments (Fig. 6B). Remarkably, FP-CATH9 maintained its high enhancement activity after treatment at 80°C and 100°C. Hence, FP-CATH9 exhibited both good serum tolerance and high thermal stability.

### Ethidium bromide accumulation assay

**Fig 7:**
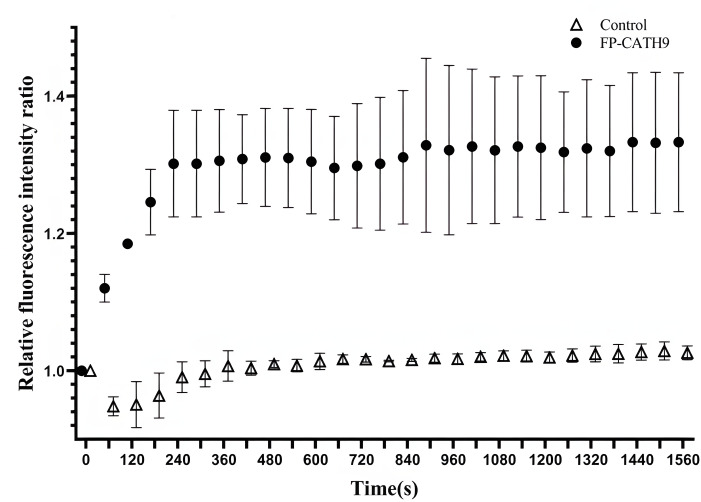
Detection of relative fluorescence intensity of ethidium bromide. Control: contains bacteria and normal saline, FP-CATH9: contains bacteria, normal saline, and FP-CATH9. Relative fluorescence intensity means FIU_(sample)_/FIU_(0s)_.

The efficiency of the intracellular efflux pump system was assessed by measuring fluorescence intensity. In this assay, the intracellular accumulation of ethidium bromide (EtBr) is a proxy for the accumulation of minocycline. When the efflux pumping system is inhibited or inactive, there is an increase in the accumulation of ethidium bromide. As shown in Fig. 7, a 24% ± 9.2% increase in the intracellular content of ethidium bromide (compared to the blank control) was observed.

### *In vivo* activity assay experiments

**Fig 8:**
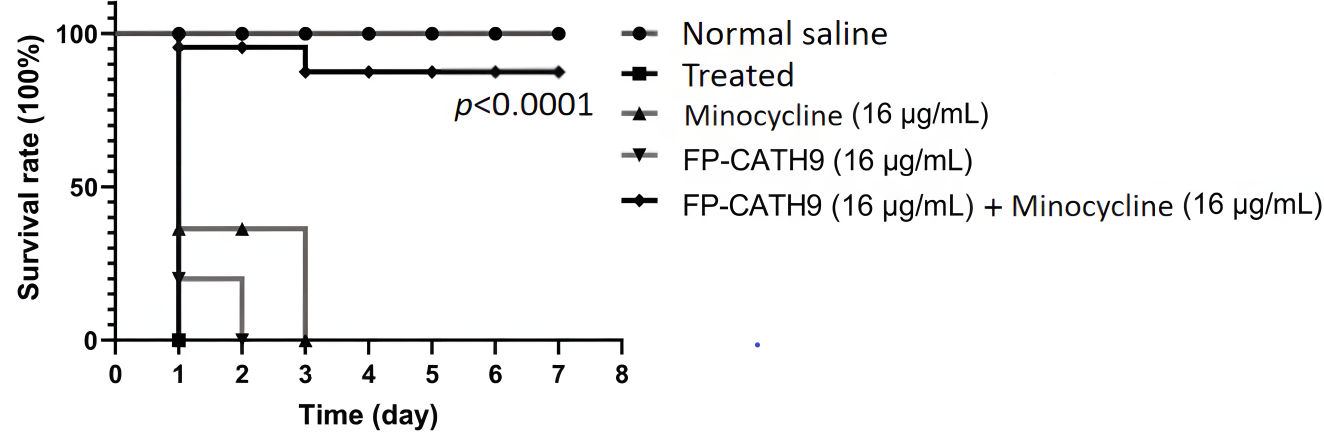
Protective effects of FP-CATH9 combined with minocycline on *G. mellonella* larvae, 10 larvaes per group, were examined for survival of *G. mellonella* larvae within 7 days.

The *G. mellonella* model of bacterial infection was used to evaluate the combined protective effect of FP-CATH9 and minocycline. In each *G. mellonella* group (*n* = 10), *G. mellonella* survival was recorded every 24 h after infection. The larvae were classified as dead when they did not respond to touch. As shown in Fig. 8, the 7 day survival rate in the uninfected saline treatment group (NS) was 100%. After the infected group was injectioned with minocycline -resistant *K. pneumoniae* strain 3 , the larvae all died within 1 day. While treatment with FP-CATH9 (16 µg/mL) alone or minocycline (16 µg/mL) alone yielded little protective effect, FP-CATH9 (16 µg/mL) + minocycline (16 µg/mL) co-treatment yielded a strong protective effect, with 80% survival at 7 days.

## DISCUSSION

AMPs are a class of small molecular peptides with a broad spectrum of antimicrobial activity. They occur naturally in organisms and play an important role in resisting pathogen invasion. In this study, we found that the cationic peptide FP-CATH9 derived from the antimicrobial peptide FP-CATH did not exhibit antibacterial activity, but it did exhibit strong sensitizing activity in combination with minocycline (and was identified during an antibiotic sensitization screening test). Minocycline is a semisynthetic tetracycline antibiotic with broad-spectrum antimicrobial activity. It inhibits bacterial protein synthesis by binding to the 30S subunit of the bacterial ribosome, precluding the attachment of aminoacyl tRNA. However, certain strains of bacteria have developed resistance to minocycline by expressing efflux pumps that help the bacteria to resist harmful substances (14, 15). These efflux pumps, for example, AdeABC and AdeIJK, have been demonstrated to pump a range of antibiotics out of host bacterial strains, including tetracycline.

To study the efflux pump systems of these bacteria, ethidium bromide accumulation assays are commonly used. This fluorescent dye is used as an indicator of efflux pump activity. For these experiments, bacterial cells were cultured in a medium containing ethidium bromide, and then the efficiency of the efflux pump was assessed by measuring fluorescence intensity inside and outside the cell. If the efflux pump system is inhibited or inactive, there is an increased accumulation of ethidium bromide. Remarkably, FP-CATH9 was found to induce the intracellular accumulation of ethidium bromide. Thus, FP-CATH9 likely inhibits the bacterial efflux pump system, leading to an accumulation of minocycline in the bacterial cells. This accumulation facilitates the inhibition of bacterial protein translation and ultimately bacterial death. Interestingly, FP-CATH9 appears to increase sensitivity to minocycline very specifically. We speculate that FP-CATH9 acts specifically on a bacterial efflux pump system that targets minocycline. However, further studies are needed to identify its specific targets and elucidate its mechanism of action in greater detail.

In the present study, FP-CATH9 was shown to increase sensitivity to minocycline in *E. coli*, *A. baumannii*, *K. pneumoniae*, and *P. aeruginosa*. FP-CATH9 was also shown to exhibit low biotoxicity and good biocompatibility. In an *in vivo* model of bacterial infections, FP-CATH9 combined with minocycline demonstrated a strong protective effect against *G. mellonella* larvae infected with multidrug-resistant *K. pneumoniae*.

In summary, we have identified a novel function for FP-CATH9 as a non-antimicrobial cationic peptide with a sensitizing effect to minocycline in multidrug-resistant gram-negative bacteria, especially minocycline-resistant bacteria. This study is of great significance for the development and utilization of antibiotic sensitizing agents, and it may help to alleviate the problem of antibiotic resistance, improve the effects of antibiotic treatment, and reduce their overall use.

## MATERIALS AND METHODS

### Bacterial and chemical agents

The strain used in this study, *E. coli* BW25113, was obtained from Yale University Escherichia coli Genetic Preservation Center, and the clinical isolate was obtained from the First Affiliated Hospital of Nanchang University. Other strains were obtained from laboratory preservation. The FP-CATH9 peptide was synthesized by Wuhan Aoke Dingsheng Biotechnology Co., Ltd., with purity of >95%. EtBr and minocycline are available in Nanchang Yingjun Technology Development Co., Ltd.

### Circular dichroism

Refer to the method of a reference (16). FP-CATH9 was dissolved in PBS buffer and 25 mM SDS (membrane simulated condition) at a concentration of 10 µM. Three scans were performed in the wavelength range of 180–260 nm.

### Screening of antibiotic sensitizing activity of FP-CATH9

The MIC of FP-CATH9 was determined using broth microdilution as recommended by Clinical and Laboratory Standards Institute (CLSI). Sensitivity to other antibiotics was assessed using the VITEK 2 Compact System (bioMerieux, France). Antimicrobial activity was determined using the Clinical and Laboratory Standards Institute (CLSI) Standard Reference method(17).

### Checkerboard assays

The synergistic activity between FP-CATH9 and minocycline, as well as the FICI, was determined by the checkerboard method (18). The two substances were mixed in 96-well plates, and then 5 × 10^5^ CFU/mL bacterial suspension was added, and the antibacterial results were observed after incubation at 37°C for 18 h. The experiment was independently repeated three times. The FICI was calculated according to the formula as follows: FICI = MIC drug A combined / MIC drug A alone + MIC drug B combined / MIC drug B alone. Based on the value of FICI, the effect of the combination of drugs can be judged. Fractional Inhibitory Concentration Index (FICI ≤0.5) indicates that the effect is significantly enhanced when the two drugs are combined. ‌The irrelevant effect‌ (0.5 < FICI ≤ 4) indicates that the combination of the two drugs has a similar effect to their individual use. ‌Antagonism‌ (FICI >4) indicates that the effects of the two drugs cancel out or diminish when used in combination.

### Diffusion method

The concentration of the FP-CATH9 mother solution was 2 mg/mL. The control conditions included MH paper + normal saline (10 µL) , experimental conditions included MH paper + FP-CATH9 (10 µL). Total 10 µL was added to MH paper to detect the dose dependence of sensitization.

### Time-killing curve

Refer to the method of a reference (19). The above clinical *K. pneumoniae* strain 3 was taken in logarithmic stage (oscillatory culture at 37°C) and diluted with fresh MH liquid medium into 10^6^ CFU/mL bacterial suspension. FP-CATH9, minocycline, and FP-CATH9 + minocycline dissolved in normal saline were added to the bacterial suspension, and the final concentration of both was 16 µg/mL. The bacterial solution added with the drug was placed in an incubator at 37°C for shock culture, and the 50 µL bacterial solution was diluted 1,000 times at 0, 2, 4, 6, 8, 10, and 12 h, respectively. Then, the 50 µL diluted bacteria solution was coated on MH solid medium, and the bacterial colonies were counted after overnight culture at 37°C. Saline was used as a negative control.

### Determination of ethidium bromide accumulation

With reference to the literature, the ethidium bromide accumulation test was slightly modified (20). In short, fresh *K. pneumoniae* strain 3 monoclonal was selected and inoculated in Luria-Bertani (LB) medium at 37°C overnight and then cultured at 1:1,000 passage. The bacteria were centrifuged at 37°C to the exponential phase, then centrifuged at 5,000 rpm for 1 min. The bacterial precipitate was suspended in normal saline, and the OD_600_ was adjusted to 0.8. Then, with only saline as a negative control, the accumulation reaction of ethidium bromide was prepared by adding 75 µL suspension cells, 15 µL ethidium bromide, 30 µL peptide or normal saline (control), and 30 µL normal saline in a 96-well microplate with a black transparent bottom. Wells containing saline and drug without cells were used as blanks to subtract the fluorescence caused by the tested compounds. The relative fluorescence unit of each hole was measured by the GloMax-Multi Detection System at 515 nm excitation and 600 nm emission at 25°C. Tests were performed in 1,560 s with three replicates per concentration, and the experiment was repeated three times.

### Hemolytic and cytotoxicity assay of FP-CATH9

Refer to the method of a reference (21). Determination of hemolytic activity. Fresh bovine blood cells (Guangzhou Hongquan Biotechnology Co., Ltd., No: HQ80074) were washed with normal saline three times and configured into 5% erythrocyte suspension. The above red blood cell suspension was mixed with FP-CATH9, FP-CATH9 + minocycline, and minocycline dissolved in normal saline at a certain concentration, incubated at 37°C for 1 h, and then centrifuged at 1,000 rpm for 5 min. The supernatant was absorbed, and the absorption value was measured at a 540 nm wavelength. Normal saline was used as negative control, and 0.2% Triton X-100 was used as positive control. The percentage of hemolysis rate is calculated by the following formula: hemolysis rate % = A(sample) − A(negative control) / A(positive control) × 100%.

### Determination of cytotoxicity

The cytotoxicity of the drug to mouse Raw264.7 cells was determined by the CCK method. FP-CATH9 and minocycline were combined according to different final concentrations. The specific steps were briefly as follows: the drug was dissolved in serum-free RPMI 1640 medium and then added to a 96-well plate (2 × 10^4^ cells/well) containing mouse Raw264.7 cells, and the serum-free RPMI 1640 medium without drug was used as a blank control. CCK-8 reagent (10 µL) was added into each well. After incubation for 4 h, the absorbance at 450 nm was measured. The results of three independent experiments were averaged, and the cell viability was calculated as % = [*A*_dosed_ − *A*_blank_] / [*A*_0-dosed_ − A_blank_] × 100%. A blank represents the absorbance of the hole containing the medium and CCK solution without cells.

### Serum and thermal stability of FP-CATH9

A single colony of activated test strain (clinical *K. pneumoniae* strain 3) was selected, diluted with normal saline to 0.5 MCG unit concentration, evenly coated with sterile cotton pads on MH solid medium plate, and placed on the surface of the medium with a minocycline drug-sensitive sheet (Thermo Fell Technology Co., Ltd.). Fetal bovine serum was purchased from Hangzhou Sijiqing Biological Engineering Materials Co. Ltd. The concentration of FP-CATH9 mother solution was 2 mg/mL. The serum stability of FP-CATH9 was determined by maintaining the total volume of 10 µL and the content of FP-CATH9 at 10 µg, and the serum concentration gradients were 0%, 6.25%, 12.5%, 25.0%, 50.0%, and 100.0%. The changes in antibacterial circle size in different groups were compared.

### Determination of the thermal tolerance of FP-CATH9

The mother liquor of FP-CATH9 was incubated in a constant temperature metal bath at 4°C, 20°C, 40°C, 60°C, 80°C, and 100°C for 1 h. After cooling, the sample solution of FP-CATH9 after incubation was 10 µL, and the sensitized effect of FP-CATH9 was determined by plate method. All plates coated with bacteria were incubated overnight at 37°C upside down.

### *G. mellonella* infection assay

Refer to the method of a reference (22). Monoclonal *K. pneumoniae* strain 3 was selected and cultivated in 5 mL LB broth using a constant temperature oscillator at 200 rpm/min and 37°C for 12 h. After 12 h, the culture was expanded and diluted at the ratio of 1:100. The logarithmic bacterial solution was taken, washed with normal saline, and then suspended in 1 mL system with a concentration of 1.5 × 10^8^ CFU/mL. The experiment was divided into 5 groups: the minocycline-resistant *K. pneumoniae* strain 3 group (treated group), the group injected with FP-CATH9 (16 µg/mL) alone, the group with minocycline (16 µg/mL) alone, the group with FP-CATH9 (16 µg/mL) + minocycline (16 µg/mL), and the NS group (saline group). After receiving 10 µL of the minocycline-resistant *K. pneumoniae* strain 3 bacterial solution (concentration 1.5 × 10^8^ cfu/mL), the drug and the bacterial solution were mixed evenly and injected into the right hind limb of the larvae immediately, and the infected larvae were incubated without food in the dark at 37°C for up to 7 days. For each group (*n* = 10), survival was monitored after infection; survival was recorded every 24 h; and death was determined when they did not respond to touch.

### Statistical analysis

All experiments were repeated three times. The results (mean ± SD) were statistically analyzed by one-way analysis of variance with repeated measures. Using GraphPad Prism version 9 software and Kaplan-Meier to calculate *G. mellonella* survival.
